# Efficacy, effectiveness and safety of vaccination against human papillomavirus in males: a systematic review

**DOI:** 10.1186/s12916-018-1098-3

**Published:** 2018-07-18

**Authors:** Thomas Harder, Ole Wichmann, Stefanie J. Klug, Marianne A. B. van der Sande, Miriam Wiese-Posselt

**Affiliations:** 1 Robert Koch Institute, Immunization Unit, Seestrasse 10, 13353 Berlin, Germany; 2 Department of Sport and Health Sciences, Technical University Munich, Chair of Epidemiology, Munich, Germany; 3 Institute of Tropical Medicine, Antwerp, Belgium

**Keywords:** Human papillomavirus, Systematic review, Efficacy, Effectiveness, Safety

## Abstract

**Background:**

Human papillomavirus (HPV) vaccination is safe and effective in preventing cervical cancer in females. As HPV infections can also induce cancers of the anus, penis and oral cavity, male vaccination is also advocated, but systematic reviews on efficacy and safety in males are lacking.

**Methods:**

We performed a systematic review on the efficacy, effectiveness and safety of HPV vaccination in males of any age. MEDLINE, Embase, the Cochrane Central Register of Controlled Trials and ClinicalTrials.gov were searched from inception to April 2017.

**Results:**

We identified 5196 articles and seven studies (four randomized controlled trials (RCTs), three non-randomized studies) were included, comprising a total of 5294 participants. Vaccine efficacy against at least 6-month persisting anogenital HPV 16 infections was 46.9% (95% confidence interval (CI) 28.6–60.8%), whereas efficacy against persisting oral infections was 88% (2–98%). A vaccine efficacy of 61.9% (21.4–82.8%) and 46.8% (− 20 to –77.9%) was observed against anal intraepithelial neoplasia grade 2 and grade 3 lesions, respectively. No meaningful estimates were available on vaccine efficacy or effectiveness against penile intraepithelial neoplasia grade 2 or 3, and no data were identified for anal, penile or head and neck squamous cell cancer. In participants who were HPV-seronegative and PCR-negative at enrolment, efficacy against all outcomes was higher as compared to seropositive and/or PCR-positive individuals. Risk of bias was low in three RCTs and high in one, while the three non-randomized studies were at serious to critical risk of bias. Grading of Recommendations Assessment, Development and Evaluation evidence quality was moderate to low for most outcomes.

**Conclusions:**

HPV vaccination in males is moderately effective against persistent anogenital HPV infection and high-grade anal intraepithelial lesions in studies where the population consists mainly of HPV-infected males. Vaccine effectiveness was high in study groups comprising HPV-naïve males. This supports a recommendation for vaccination of boys before the onset of sexual activity with the goal of establishing optimal vaccine-induced protection. Mathematical modelling studies will still be needed to assess the effects of adding males to existing HPV vaccination programs in females.

**Trial registration:**

Prospective Register for Systematic Reviews (PROSPERO) registration CRD42016038965.

**Electronic supplementary material:**

The online version of this article (10.1186/s12916-018-1098-3) contains supplementary material, which is available to authorized users.

## Background

Human papillomavirus (HPV) is the most common sexually transmitted microorganism. The skin and mucosa of the anogenital tract, oral cavity, oropharynx and larynx are frequently affected by this virus [1]. The initial HPV infection is asymptomatic, and the virus is cleared in the majority of cases [2, 3]. About 10% of HPV infections persist, and less than 3% result in epithelial dysplasia or even cancer (1%) if the infection is due to an oncogenic HPV type [4–6]. Presently, more than 200 different HPV types have been described. They are classified as high risk (HR) and low risk (LR) viruses. While LR types such as HPV 6 and HPV 11 can cause genital warts, infections due to HR HPV types can induce cancer. The International Agency for Research on Cancer (IARC) classifies 12 HPV types as HR cancer-causing types, of which HPV 16 and HPV 18 are the most common [7, 8]. In females, nearly 100% of cervical cancers are attributable to HR HPV types [9]. In males, approximately 33% of penile cancers and up to 90% of anal cancers are attributed to HR HPV infections, primarily with HPV type 16 [10–12]. The HPV attributable fraction in cancers of the oral cavity, oropharynx and larynx was estimated to be 22.4%, 4.4% and 3.5%, respectively [13].

Anogenital HPV infections are common in men. In a population-based survey in the USA, 23% of participants had a penile infection with a high-risk HPV type [14]. In men who have sex with men (MSM) and in HIV-positive men, even higher prevalences were observed. A Dutch study reported that 45% of HIV-negative MSM and 65% of HIV-positive MSM had an anal infection by a high-risk HPV type [15]. HPV infections of the oral cavity are less prevalent: a systematic review reported a pooled prevalence of 4.5%, with no significant differences between men and women [16]. According to the German Centre for Cancer Registry Data, in 2013 a total of 1358 male cancer cases in Germany were attributable to HPV [17]. The life-time risk of HPV-associated genital warts has been estimated to be 5–10% [18].

To date, three different vaccines against HPV have been licensed [19]. They contain virus-like particles (VPLs) that induce immunity against certain HPV types. The bivalent vaccine protects against HR HPV types 16 and 18, and the quadrivalent vaccine protects against HR HPV types 16 and 18 as well as LR HPV types 6 and 11. A nonavalent vaccine was approved by the US and European regulatory authorities in 2015 and 2016, respectively, adding protection against five additional HR HPV types. Based on epidemiological data, it has been estimated that 85–90% of all cervical cancer cases could be prevented by vaccination with the nonavalent HPV vaccine if it is administered to girls before their sexual debut [20]. By targeting HPV 16 and 18 alone, 60–70% of all cervical cancers could be prevented.

While most industrialized countries have introduced routine female HPV vaccination into their national immunization programs, routine vaccination of boys and men is currently implemented in only a few countries, such as Australia, Canada, the USA and Austria. Vaccination of boys and men may further reduce the incidence of cervical cancer and its precursors via herd protection, and reduce the incidence of anal and penile as well as head and neck cancers [21, 22]. The aim of this systematic review was to assess the currently available evidence on the efficacy, effectiveness and safety of HPV vaccination in males.

## Methods

### Search strategy and selection criteria

This systematic review followed a protocol published in the Prospective Register for Systematic Reviews (PROSPERO; registration no. CRD42016038965) and was reported according to the guidelines in the Preferred Reporting Items for Systematic Reviews and Meta-analyses (PRISMA) statement [23]. To be eligible, a study had to investigate the efficacy, effectiveness and/or safety of vaccination (with a licensed vaccine) against HPV in males of any age. The control group had to be males who were either unvaccinated or had received placebo or a vaccine not directed against HPV. An eligible study had to report on at least one of the following predefined outcomes: (1) incident oral infection with an HR HPV type; (2) incident anogenital (or anal) infection with an HR HPV type; (3) persisting oral infection with an HR HPV type (≥ 6 months); (4) persisting anogenital (or anal) infection with an HR HPV type (≥ 6 months); (5) condyloma acuminatum (genital or anal) due to HPV 6 or 11; (6) anal intraepithelial neoplasia (AIN) grade 2; (7) AIN grade 3 or carcinoma; (8) penile intraepithelial neoplasia (PIN) grade 2; (9) PIN grade 3 or carcinoma; (10) squamous cell carcinoma of the head and neck region, including the oropharynx, larynx and oral cavity; (11) epithelial dysplasia related to (10); and (12) any severe adverse event following immunization. As we were primarily interested in clinical relevance, we did not restrict ourselves to vaccine-type specific lesions only.

The electronic databases searched were MEDLINE, Embase and the Cochrane Central Register of Controlled Trials (date of initial search 4 November 2016; last update 18 April 2017). For details on the complete search strategy, see Additional file 1. Additionally, ClinicalTrials.gov was searched systematically for unpublished or ongoing trials. Electronic searches were complemented by manually screening conference abstract books of major international HPV conferences (EUROGIN 2016, International HPV Conference 2017) as well as reference lists of all identified studies and those of identified reviews. Search results (titles, abstracts, full texts) were independently assessed by two investigators (TH, MWP). Differences were discussed until a consensus was reached.

We did not make any restrictions with regard to setting, language or publication status (published/unpublished). Potential indirect effects of male HPV vaccination on the incidence of clinical outcomes in females were not considered in this review.

### Data extraction

From the eligible studies, two independent reviewers (TH, MWP) used standardized forms to extract study characteristics and assess methodological quality. In case of disagreement, a final decision was made by consensus. The corresponding authors or principal investigators of three studies were contacted for additional data and information [24–26]. The following data were extracted: study location, study design, study period, inclusion criteria, exclusion criteria, age at enrolment, duration of follow-up, vaccine used, comparator, study sponsorship, conflict of interests, number (proportion) of vaccinated participants with outcome, number (proportion) of control participants with outcome, unadjusted estimates, adjusted estimates, confounders.

### Assessment of risk of bias and quality of evidence

For randomized controlled trials (RCTs), the Cochrane risk of bias tool was used to assess the following domains: random sequence generation, allocation concealment, blinding of participants and personnel, blinding of outcome assessment, incomplete outcome data, selective reporting, other bias [27]. Studies were categorized as being at “high risk”, “low risk” or “unclear risk” of bias. For non-randomized studies, the Risk Of Bias in Non-randomized Studies - of Interventions (ROBINS-I) tool was used, comprising the following domains: bias due to confounding, bias in selection of participants into the study, bias in classification of interventions, bias due to deviations from intended interventions, bias due to missing data, bias in measurement of outcomes, bias in selection of the reported results [28]. Risk of bias was categorized as being “low risk”, “moderate risk”, “serious risk” or “critical risk”.

The methodology of the Grading of Recommendations, Assessment, Development and Evaluation (GRADE) working group was used to assess the quality of the evidence [29, 30].

### Statistical analysis

Abstracted data were aggregated in tables. Risk ratios, odds ratios, risk differences and corresponding 95% confidence intervals (95% CI) were either calculated or extracted from the publications. Vaccine efficacy and vaccine effectiveness were either extracted from the publications or calculated as [1-(risk ratio or rate ratio comparing vaccine and control recipients)] × 100. Since only one study per outcome and study design was identified in this review, no meta-analyses were performed. According to the review protocol, analyses were performed in two subgroups: (1) all study participants, irrespective of HPV infection at enrolment, and (2) study participants who were seronegative and PCR-negative for the relevant HPV types at enrolment.

## Results

**Fig. 1:**
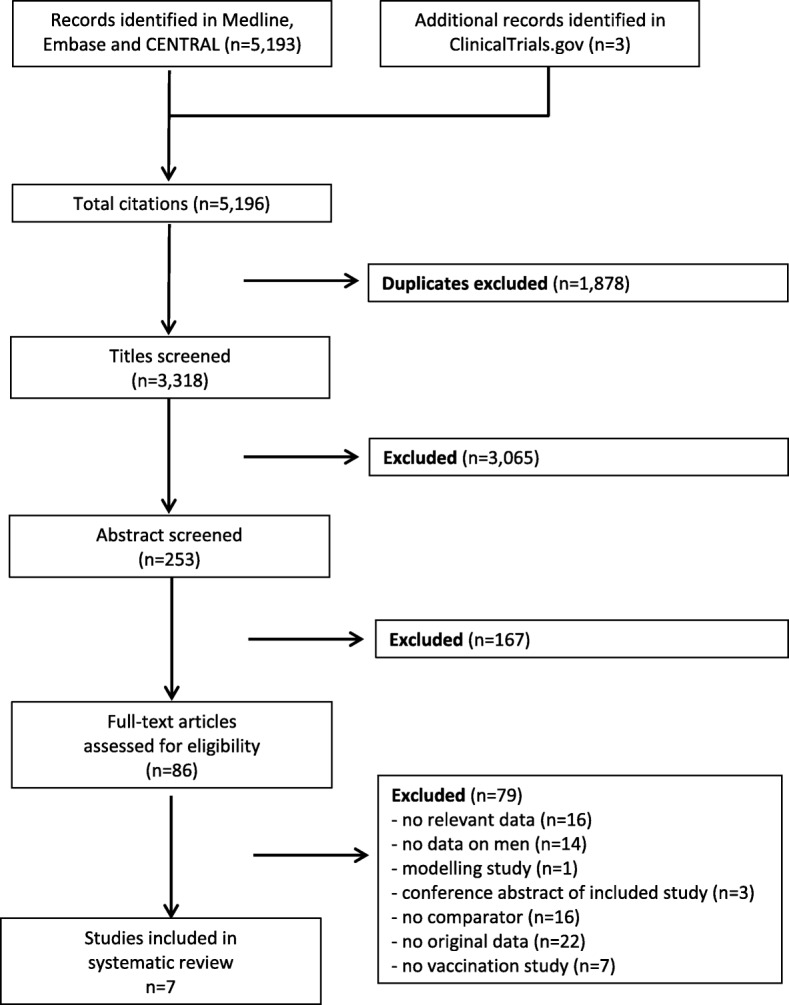
Flow diagram

We identified a total of 5196 entries in electronic databases. After exclusion of duplicates, 3318 articles remained for title screening. Of these, 3065 papers were judged to be irrelevant according to their titles and were excluded. The abstracts of the remaining 253 publications were subsequently screened, and 167 of them were considered irrelevant and therefore excluded. We then assessed the full text of the remaining 86 articles. Of these, 79 were excluded due to lack of relevant data (*n* = 16), lack of data on men (*n* = 14), being a modelling study (*n* = 1), being a conference abstract of an included study (*n* = 3), lack of comparator (*n* = 16), lack of original data (*n* = 22) or not being a vaccination study (*n* = 7). Thereby, we finally included seven studies in the analysis (for details, see Fig. 1). Bibliographic data of the 79 studies that were excluded after full-text assessment are reported in Additional file 2.

We included four RCTs [26, 31–33] and three non-randomized studies [24, 25, 34]. The studies comprised data of a total of 5294 study participants. One included RCT [33] was a subtrial of another included study [32], but it reported different outcomes. The main study characteristics are shown in Table 1. Three of these trials were international multicentre studies [26, 32, 33], while the remaining four studies were performed solely in the USA or in Turkey. Four of the studies included both heterosexual men and MSM [25, 32–34]. One study included only self-identified heterosexual men [31]. The other two studies recruited male and female participants, but only the results for males are reported here. One of these studies was performed in adolescents and young adults with behaviorally acquired HIV [24], whereas the other one included HIV-positive adults [26]. The age of the participants at study entry ranged from 12 to 76 years. In all studies, the quadrivalent vaccine (Gardasil) was used. Six studies provided information on potential conflicts of interest [24–26, 32–34]. Two studies were registrative studies and reported sponsorship by a pharmaceutical company [32, 33].

**Table 1:** Characteristics of included studies

Study	Location	Design	Study period	Inclusion criteria	Exclusion criteria	Age at enrolment (median (range))	Duration of follow-up (median)	Vaccine/comparator	Study sponsorship	Conflict of interest	Number of participants	Outcomes
Giuliano et al. 2011 [32]	Multicentre (18 countries)	RCT	2004–ongoing (enrolled until 08/2008)	Heterosexual men 16–23 years of age who had 1–5 life-time sex partners or MSM 16–26 years. of age who had 1–5 male or female life-time sex partners	Men with clinically detectable anogenital warts or genital lesions or history of such findings	20 years. (16–26 years)	2.9 yrs	Gardasil/placebo	Merck; National Institutes of Health	Merck, GlaxoSmith-Kline (GSK), Qiagen, AstraZeneca	Vaccinated: 2032; placebo: 2033	PIN grade 2 or 3; penile, perineal or perianal cancer; persistent anogenital infection; incident anogenital infection; genital condyloma acuminatum; severe adverse events
Palefsky et al. 2011 [33]	Multicentre (7 countries)	RCT (substudy of Giuliano et al. 2011 [32])	2004– ongoing (enrolled until 08/2008)	MSM 16–26 years. of age who had 1–5 male or female life-time sex partners and were engaged in insertive or receptive anal intercourse with another man in the past year	Men with history or presence of clinically detectable anogenital warts or genital lesions or intra-anal lesion on anoscopy consistent with AIN or condyloma	22 years. (16–26 years)	2.2 years	Gardasil/placebo	Merck; National Institutes of Health	Merck, GSK, Qiagen, Pharmajet, Aura Biosciences, Roche, GenProbe	Vaccinated: 301; placebo: 301	AIN grade 2; AIN grade 3; anal cancer; persistent anal infection; incident anal infection; anal condyloma accuminatum; severe adverse events
Swedish et al. 2012 [25]	USA	Cohort study	2007–2011	MSM 18 years. or older with a history of biopsy-proven and treated high-grade AIN	Less than three vaccine doses; high-grade AIN at study start	40.4 years. (20.3–72.3 years)	Vaccinated: 489 days; unvaccinated: 722 days	Gardasil/unvaccinated	NR	Merck, Qiagen, Asha	Vaccinated: 88; unvaccin-ated: 114	Recurrent high-grade AIN (AIN grade 2 or 3)
Coskuner et al. 2014 [31]	Turkey	RCT (not placebo-controlled)	2009–2013	Men with new onset genital warts living in the same area for at least 1 year	Previous treatment for preexisting warts; medical disorders that needed chronic treatment; immune suppression (incl. HIV)	Mean: 34 years. (+/− 7.6)	4 years after first dose	Gardasil/unvaccinated	NR	NR	Vaccinated: 91; unvaccinated: 80	Recurrent condyloma acuminatum
Swedish and Goldstone 2014 [34]	USA	Cohort study	2007–2013 (vaccinated until 2010)	Self-identified MSM, HIV-negative, no prior history of anal condyloma or previously treated anal condyloma but recurrence-free for at least 12 months prior to study entry	Men with anal or penile condyloma at study start	42.2 years. (range 26.1–76 years)	Up to 4 years. (vacc: median 880 days; unvacc: median 1039 days)	Gardasil/unvaccinated	None	Merck	Vaccinated: 116; unvaccinated: 197	Anal condyloma
Kahn et al. 2015 [24]	USA (5 centres)	Cross-sectional study	2011–2012	Youth with behaviorally acquired HIV	Serious psychiatric illness, intoxication with alcohol or drugs	21.5 years. (range 12–24 years)	NA	Gardasil/unvaccinated	National Institutes of Health	Merck	Vaccinated: 23; unvaccinated: 48	(Incident) oral infection (HPV 16 and/or HPV 18)
NCT01461096 [26, 47]	USA, Brazil	RCT	2012–2015 (stopped due to futility)	HIV-infected adults	History of cancer, history of AIN, previous HPV vaccination, anticoagulation, allergies, drug/alcohol dependence, serious illness, immunomodulatory treatment; hepatitis B/C treatment	47 years (intraquartile range: 41–53)	2.1 years. (median)	Gardasil/placebo	National Institute of Allergy and Infectious Diseases	Gilead, Bristol Meyer Squibb, GSK	Vaccinated: 236; placebo: 236	Persisting oral infection^a^

*AIN* anal intraepithelial neoplasia, *MSM* men who have sex with men, *NA* not applicable, *NR* not reported, *PIN* penile, perineal or perianal neoplasia, *RCT* randomized controlled trial

^a^Data on other outcomes available for combined analysis of males and females

Only two of the studies reported data on HPV-naïve participants [32, 33]. These studies were registrative trials and comprised a total of 2032 participants in the vaccinated and 2033 participants in the placebo arm. Of those, 1397 subjects in the vaccinated and 1408 in the placebo group were HPV-naïve men. The mean age in the vaccinated group was 20.6 years (standard deviation (SD) 2.0; range 16–26 years); it was 20.5 years (SD 2.0; range 15–27 years) in the placebo group. The median length of follow-up was 2.9 years for both groups.

Of the 12 outcomes defined a priori in the review protocol, ten were reported in the included studies (Table 2). In two of the RCTs [32, 33], the primary efficacy endpoints defined by the investigators were compound endpoints comprising malignant and benign anal or genital lesions, but only those data that correspond to the outcomes predefined in our protocol were used for the following analyses. We considered the outcome “DNA detection” reported in these two trials [32, 33] as being equivalent to “incident infection”. Furthermore, because different sampling techniques were used, we separated “anal infection” from “anogenital infection” in these two studies [32, 33]. A total of four studies reported data on condyloma acuminatum, while two studies reported on the outcomes AIN grade 2, AIN grade 3 and/or severe adverse events. For the remaining outcomes, only one study per outcome was identified. We did not identify any relevant studies investigating the outcomes 10 “squamous cell carcinoma of the head and neck region” or 11 “epithelial dysplasia related to squamous cell carcinoma of the head and neck region”.

**Table 2:** Overview of outcomes reported in the studies

Study	Incident oral infection	Persisting oral infection	Incident anogenital infection		Persisting anogenital infection		Condyloma acuminatum		AIN grade 2	AIN grade 3 or carcinoma	PIN grade 2	PIN grade 3 or carcinoma	Severe adverse events
			Anogenital	Anal	Anogenital	Anal	Genital	Anal					
Randomized trials													
Giuliano et al. 2011 [32]			x		x		x				x^a^	x^a^	x
Palefsky et al. 2011 [33]				x		x		x	x	x			x^b^
Coskuner et al. 2014 [31]							x						
NCT01461096 [26]		x											
Non-randomized studies													
Swedish et al. 2012 [25]									x^c^	x^c^			
Swedish and Goldstone 2014 [34]								x					
Kahn et al. 2015 [24]	x												

^a^PIN 2 and PIN 3 analysed together

^b^Subgroup of Giuliano et al. [32]; therefore, all severe adverse events already reported there

^c^defined as “high-grade anal intraepithelial neoplasia”, comprising AIN 2 and AIN 3 (Goldstone, personal communication). Applying a conservative approach, results are reported here as AIN 2

Risk of bias was low in three of the RCTs (Table 3). In the remaining RCT [31], risk of bias was judged to be high due to the lack of a placebo-controlled study arm (controls were unvaccinated). The three non-randomized studies were judged to have a serious (two studies) or critical risk of bias (one study) due to residual confounding, risk of selection bias and missing data (Table 4).

**Table 3:** Risk of bias in randomized controlled trials

Study	Random sequence generation	Allocation concealment	Blinding of participants and personnel	Blinding of outcome assessment	Incomplete outcome data	Selective reporting	Other bias
Giuliano et al. 2011 [32]	+	+	+	+	+	+	+
Palefsky et al. 2011 [33]	+	+	+	+	+	+	+
Coskuner et al. 2014 [31]^a^	+	+	–	–	+	+	+
NCT01461096 [26]	+	+	+	+	+	+	+

+ low risk of bias, − high risk of bias

^a^Controls received no vaccination, yet blinding was not possible

**Table 4:** Risk of bias in non-randomized studies

Study	Confounding	Selection bias	Classification of intervention	Deviation from intervention	Missing data	Outcome measurement	Selection of reported results	Overall risk of bias
Swedish et al. 2012 [25]^a^	+	–	++	++	–	++	++	–
Swedish and Goldstone 2014 [34]^a^	+	–	++	++	–	++	++	–
Kahn et al. [24]^b^	– –	–	–	++	–	++	++	– –

++ low risk of bias, + moderate risk of bias, − serious risk of bias, – –- critical risk of bias

^a^Unclear selection into study; differences in follow-up time between groups; loss-to-follow up

^b^Only unadjusted data available; unclear selection into study; loss-to-follow up; possible misclassification of intervention

Table 5 shows vaccine efficacy and effectiveness against infection with HR HPV types (HPV 16 or HPV 18), irrespective of HPV infection status at enrolment. Vaccine efficacy was low against incident anogenital infections caused by HPV 16 (28%) and HPV 18 (33.9%), but was higher against incident anal infections (45.1% against HPV 16; 49.5% against HPV 18), although the 95% CIs were overlapping. Vaccine efficacy estimates for preventing persisting (defined as ≥ 6 months) anogenital and anal infections were higher than those for incident infections (46.9–73.6%). Two of the RCTs also reported vaccine efficacy in participants who were seronegative and PCR-negative for the respectively studied HPV types at enrolment. As shown in Table 6, vaccine efficacy estimates were higher for all outcomes compared with those in the analyses performed irrespective of HPV status, ranging from 41.1% (incident anogenital infection with HPV 16) to 100% (incident and persisting anal infection with HPV 18), with wide confidence intervals.

**Table 5:** Efficacy or effectiveness of vaccination against human papillomavirus in males: infections in participants irrespective of their HPV status at enrolment

Study	Design	No. of events/no. of participants		Unadjusted estimate (95% CI)	Confounder-adjusted estimate (95% CI)	VE (95% CI)
		Vaccine	Control			
Incident anogenital infection						
*HPV 16*						
Giuliano et al. 2011 [32]^a^	RCT	189/4070.0 pyrs	259/4014.2 pyrs	NR	NA	28.0% (12.9–40.7%)
*HPV 18*						
Giuliano et al. 2011 [32]^a^	RCT	89/4205.4 pyrs	133/4151.5 pyrs	NR	NA	33.9% (13.0–50.1%)
Persisting anogenital infection						
*HPV 16*						
Giuliano et al. 2011 [32]^a^	RCT	71/4199.5 pyrs	131/4112.7 pyrs	NR	NA	46.9% (28.6–60.8%)
*HPV 18*						
Giuliano et al. 2011 [32]^a^	RCT	25/4267.0 pyrs	56/4210.1 pyrs	NR	NA	56.0% (28.8–73.7%)
Incident anal infection						
*HPV 16*						
Palefsky et al. 2011 [33]^a^	RCT	40/615.7 pyrs	71/599.9 pyrs	NR	NA	45.1% (18.0–63.7%)
*HPV 18*						
Palefsky et al. 2011 [33]^a^	RCT	20/651.2 pyrs	39/641.3 pyrs	NR	NA	49.5% (11.3–72.1%)
Persisting anal infection						
*HPV 16*						
Palefsky et al. 2011 [33]^a^	RCT	24/636.6 pyrs	51/622.3 pyrs	NR	NA	54% (23.9–72.9%)
*HPV 18*						
Palefsky et al. 2011 [33]^a^	RCT	7/668.4 pyrs	26/656.3 pyrs	NR	NA	73.6% (37.5–90.3%)
Incident oral infection						
*HPV 16 and/or HPV 18*						
Kahn et al. 2015 [24]^a^	Cross-sectional study	0/23	9/48	NR	NR	91% (−59–99.5%)
Persisting oral infection						
NCT01461096 [26]^b^	RCT	1/236	8/236	0.12 (0.02–0.98)^c^	NR	88% (2–98%)

*NA* not applicable, *NR* not reported, *pyrs* person-years, *VE* vaccine efficacy or effectiveness

^a^VE as reported in the primary study

^b^VE calculated from unadjusted estimate

^c^Hazard ratio (95% CI)

**Table 6:** Efficacy or effectiveness of vaccination against human papillomavirus in males: infections in participants who were seronegative and PCR-negative at enrolment

Study	Design	No. of events/no. of participants		Unadjusted estimate (95% CI)	Confounder-adjusted estimate (95% CI)	VE (95% CI)
		Vaccine	Control			
Incident anogenital infection						
*HPV 16*						
Giuliano et al. (2011) [32]^a^	RCT	62/2337.7 pyrs	103/2287.8 pyrs	NR	NA	41.1% (18.5–57.7%)
*HPV 18*						
Giuliano et al. (2011) [32]^a^	RCT	25/2441.3 pyrs	66/2440.6 pyrs	NR	NA	62.1% (39.2–77.1%)
Persisting anogenital infection						
*HPV 16*						
Giuliano et al. (2011) [32]^a^	RCT	9/2382.4 pyrs	41/2312.9 pyrs	NR	NA	78.7% (55.5–90.9%)
*HPV 18*						
Giuliano et al. (2011) [32]^a^	RCT	1/2461.9 pyrs	25/2453.5 pyrs	NR	NA	96% (75.6–99.9%)
Incident anal infection						
*HPV 16*						
Palefsky et al. (2011) [33]^a^	RCT	6/326 pyrs	25/322.8 pyrs	NR	NA	76.2% (40.7–92%)
*HPV 18*						
Palefsky et al. (2011) [33]^a^	RCT	0/346.3 pyrs	16/375.1 pyrs	NR	NA	100% (71.9–100%)
Persisting anal infection						
*HPV 16*						
Palefsky et al. (2011) [33]^a^	RCT	1/331.5 pyrs	16/329.9 pyrs	NR	NA	93.8% (60.0–99.9%)
* HPV 18*						
Palefsky et al. (2011) [33]^a^	RCT	0/346.3 pyrs	10/376.2 pyrs	NR	NA	100% (51.5–100%)

*NA* not applicable, *NR* not reported, *pyrs* person-years, *VE* vaccine efficacy or effectiveness

^a^VE as reported in the primary study

Incident oral infections with HR HPV types were investigated in only one study, which used a non-randomized design. Here, a vaccine effectiveness of 91% was observed; however, the 95% CI was very wide and no confounder-adjusted estimate was reported. For persistent oral HPV infections, an efficacy of 88% was reported in one RCT (Table 5).

Estimates of vaccine efficacy and effectiveness against anogenital lesions are shown in Tables 7 and 8. Efficacy against genital condyloma acuminatum was investigated in two of the RCTs: one RCT reported a vaccine efficacy of 67.2%, whereas the other one did not show a protective effect (− 26%). However, the latter RCT included only participants with a history of condyloma and had a high risk of bias. Efficacy and effectiveness against anal condyloma was assessed in one RCT and in one non-randomized study. Both studies reported very similar results (57.2% and 55%, respectively), but their confidence intervals were wide. Vaccine efficacy against AIN grade 2 was reported to be 61.9% in one RCT, while vaccine effectiveness was slightly lower in a non-randomized study (50%). AIN grade 3 was investigated in only one RCT, which reported a non-significant efficacy of 46.8%. Likewise, PIN grade 2 or 3 was reported in one RCT, but the number of cases was too small in both the vaccinated (*n* = 3) and placebo groups (*n* = 2) to generate a meaningful estimate of vaccine efficacy. Since no cases of anal cancer or penile/perineal/perianal cancer were observed in the included studies, efficacy or effectiveness against these outcomes could not be calculated. Table 8 shows the respective estimates for those RCT participants who were HPV-negative at study entry [32, 33]. In this subgroup, estimates of vaccine efficacy for the prevention of anogenital lesions were higher than among individuals irrespective of HPV status, but the case numbers were small and did not lead to meaningful efficacy estimates against AIN (grade 2: efficacy 75.8%, 11 cases; grade 3: efficacy 63.7%, 8 cases), PIN (grade 2 or 3: efficacy 100%, 1 case) or cancer (no cases).

**Table 7:** Efficacy or effectiveness of vaccination against human papillomavirus in males: anogenital lesions in participants irrespective of their HPV status at enrolment (corresponding to intention-to-treat analysis in RCTs)

Study	Design	No. of events/no. of participants		Unadjusted estimate (95% CI)	Confounder-adjusted estimate (95% CI)	VE (95% CI)
		Vaccine	Control			
Condylomata acuminata						
*Genital*						
Giuliano et al. 2011 [32]^a^	RCT	24/4635.4 pyrs	72/4558.8 pyrs	NR	NA	67.2% (47.3–80.3%)
Coskuner et al. 2014 [31]^b^	RCT	45/91	35/80	1.26 (0.69–2.30)	NR	−26% (− 130 to 31%)
*Anal*						
Palefsky et al. 2011 [33]^a^	RCT	13/651.3 pyrs	31/664.2 pyrs	NR	NA	57.2% (15.9–79.5%)
Swedish & Goldstone 2014 [34]^d, e^	Cohort study	10/269.3 pyrs	37/604.3 pyrs	0.49 (0.24–0.98)	0.45 (0.22–0.92)	55% (8–78%)
Anal intraepithelial neoplasia grade 2						
Palefsky et al. 2011 [33]^a^	RCT	11/668 pyrs	29/671.5 pyrs	NR	NA	61.9% (21.4–82.8%)
Swedish et al. 2012 [25]^c, e^	Cohort study	12/117.6 pyrs	35/222.8 pyrs	0.52 (0.27–1.0)	0.50 (0.26–0.98)	50% (2–74%)
Anal intraepithelial neoplasia grade 3						
Palefsky et al. 2011 [33]^a^	RCT	10/665.9 pyrs	19/672.8 pyrs	NR	NA	46.8% (−20 to 77.9%)
Anal cancer						
Palefsky et al. 2011 [33]	RCT	0/678.4 pyrs	0/694.8 pyrs	NR	NA	–
Penile, perineal or perianal neoplasia grade 2 or 3						
Giuliano et al. 2011 [32]^a^	RCT	3/4663.1 pyrs	2/4628.6 pyrs	NR	NA	−48.9% (− 1682.6 to 82.9%)
Penile, perineal or perianal cancer						
Giuliano et al. 2011 [32]	RCT	0/4670.6 pyrs	0/4630.5 pyrs	NR	NA	–

^a^VE as reported in the primary study

^b^Recurrent lesions

^c^All participants with a history of high-grade AIN (data for 24-month follow-up period); VE calculated from unadjusted estimate

^d^103/313 participants with recurrent lesions

^e^VE calculated from confounder-adjusted estimate

*NA* not applicable, *NR* not reported, *pyrs* person-years, *VE* vaccine efficacy or effectiveness

**Table 8:** Efficacy or effectiveness of vaccination against human papillomavirus in males: anogenital lesions in participants who were seronegative and PCR-negative at enrolment

Study	Design	No. of events/no. of participants		Unadjusted estimate (95% CI)	Confounder-adjusted estimate (95% CI)	VE (95% CI)
		Vaccine	Control			
Condylomata acuminata						
*Genital*						
Giuliano et al. (2011) [32]^a^	RCT	3/2830.9 pyrs	28/2813.9 pyrs	NR	NA	89.4% (65.5–97.9%)
*Anal*						
Palefsky et al. (2011) [33]^a^	RCT	0/386.8 pyrs	6/418.2 pyrs	NR	NA	100% (8.2–100%)
Anal intraepithelial neoplasia grade 2						
Palefsky et al. (2011) [33]^a^	RCT	2/384.5 pyrs	9/418.6 pyrs	NR	NA	75.8% (−16.9 to 97.5%)
Anal intraepithelial neoplasia grade 3						
Palefsky et al. (2011) [33]^a^	RCT	2/385.4 pyrs	6/419.7 pyrs	NR	NA	63.7 (−103 to 96.4%)
Anal cancer						
Palefsky et al. (2011) [33]	RCT	0/386.8 pyrs	0/421.1 pyrs	NR	NA	–
Penile, perineal or perianal neoplasia grade 2 or 3						
Giuliano et al. (2011) [32]^a^	RCT	0/2833.3 pyrs	1/2824.7 pyrs	NR	NA	100% (− 3788.2 to 100%)
Penile, perineal or perianal cancer						
Giuliano et al. (2011) [32]	RCT	0/2833.3 pyrs	0/2826.2 pyrs	NR	NA	–

*NA* not applicable, *NR* not reported, *pyrs* person-years, *VE* vaccine efficacy or effectiveness

^a^VE as reported in the primary study

Severe adverse events following immunization were reported in two of the included RCTs [32, 33]. Because the smaller [33] was composed of a subgroup of participants from the larger RCT [32], all severe adverse events reported in the former had already been included and reported in the latter. Therefore, only data from the larger RCT were considered here. During the entire study period, 8 adverse events were observed in the vaccinated group (2020 participants) and 11 events occurred in the placebo group (2029 participants). This finding corresponds to a risk ratio of 0.73 (95% CI 0.25–1.99) and a risk difference of − 0.2 (95% CI −0.7 to 0.3). According to the trial investigators, none of these adverse events were judged to be vaccine-related.

Evidence quality according to GRADE was judged “high” only for the outcome condyloma acuminatum. For five of the included outcomes (incident anogenital infection, persisting oral infection, persisting anogenital infection, AIN grade 2 and severe adverse events following immunization), evidence quality was downgraded to “moderate” due to serious imprecision (wide 95% CIs). The outcome AIN grade 3 was judged to provide “low” quality evidence due to very serious imprecision (very wide 95% CI). Incident oral infection was assessed to provide “very low” quality evidence due to the non-randomized study design and serious imprecision. The evidence quality for PIN grade 2/3 was judged to be “very low” due to serious indirectness (the outcome in the trial comprised penile, perineal and perianal neoplasia) and very serious imprecision (see the GRADE evidence profile in Additional file 3 for details).

## Discussion

This systematic review of randomized and non-randomized studies evaluated the efficacy, effectiveness and safety of HPV vaccination in males. When vaccinating individuals irrespective of their HPV status, vaccination is moderately effective against genital HPV infection and high-grade anal intraepithelial lesions. Higher vaccine efficacy was observed in those participants who were naïve for the respective HPV types assessed in the individual studies. No meaningful estimate of vaccine efficacy could be calculated for high-grade penile intraepithelial lesions, and no data were available regarding vaccine efficacy or effectiveness against anal, penile or head and neck squamous cell cancer. Due to their imprecision of estimates, the GRADE evidence quality was moderate to low for the majority of outcomes.

To our knowledge, this is the first systematic review on the efficacy, effectiveness and safety of HPV vaccination in males. In contrast, at least seven systematic reviews of studies assessing the effects of HPV vaccination in females have been published demonstrating high efficacy and effectiveness of HPV vaccination against infection and dysplasia, particularly in HPV-naïve study participants [35–41]. Considerable differences exist in the body of evidence between male and female HPV vaccination regarding the primary study base. While our current systematic review comprised data from about 5000 participants, randomized trials on HPV vaccination in females included more than 46,000 participants in total [41]. Beyond study size, considerable differences exist between HPV trials in males and females regarding the validity of outcomes and evidence quality. For precancerous lesions of the cervix (cervical intraepithelial neoplasia), robust data from the above-mentioned systematic reviews show that vaccination against HPV prevents high-grade lesions over a time period of more than 6 years. As shown in our systematic review, the evidence base is weaker regarding precancerous lesions in males, particularly for penile lesions. Furthermore, evidence on efficacy, effectiveness and safety of HPV vaccination in males is currently restricted to a period of 4 years after vaccination. Similar to findings from HPV vaccination in females, a considerably higher vaccine efficacy has been found in men who were seronegative and PCR-negative at study entry [41].

Our systematic review has several strengths. It was conducted based on a comprehensive and published review protocol, and internationally accepted tools for the assessment of risk of bias were applied. We performed an outcome-specific assessment of the available data and assessed the quality of the body of evidence for each outcome using the GRADE methodology. We performed a comprehensive review by including different study designs and populations (MSM as well as heterosexual men). The limitations of this review mainly arise from the limitations of the included primary studies. The two major RCTs included here were designed to evaluate compound endpoints comprising malignant and benign lesions. Consequently, neither of these studies had enough power to detect premalignant or malignant lesions as defined in our protocol. Furthermore, the existing non-randomized studies have a high risk of bias. A limitation of our systematic review stems from the restriction of the outcomes to clinical endpoints. For example, we did not search for immunogenicity data and can therefore not draw conclusions from studies that used them as surrogate markers for protection. A further limitation of our systematic review may arise from the decision to focus on all lesions rather than on type-specific lesions. This approach might lead to an underestimation of the efficacy and effectiveness of the vaccine. However, our approach was chosen since it is likely to consider clinical impact and the patient perspective more appropriately than an approach that focusses on type-specific lesions. For future updates of this review, several strategies might be tested in order to improve the specificity of the search results. One could, for instance, use the “NOT” operator to exclude certain types of articles that do not contain data (such as comments or editorials) from the search. Furthermore, it appears possible to focus the search on certain study types (such as RCTs, cohort studies, case-control studies) that are relevant for the PICO (Patient, problem or population; Intervention; Comparison, control or comparator; Outcome) question.When HPV status prior to vaccination is not considered, efficacy of HPV vaccination in males is moderate, particularly regarding incident infections, and the corresponding confidence intervals are wide. In cases where only HPV-naïve participants are vaccinated, the vaccine efficacy estimates are higher, but the corresponding confidence intervals are still wide. These wide confidence intervals might be due at least partly to variations in sampling techniques, because the sampling in men has not been as standardized as it is in women [42], and the main results on genital and anal infections came from multicentre studies [32, 33]. However, the estimates reported here might be conservative, given that data from females suggest that the efficacy of HPV vaccination increases with longer follow-up due to the effect on incident but not prevalent infections [43].

Evidence on efficacy and effectiveness of HPV vaccination in males is particularly scarce regarding oral infections; only one RCT with a small number of events and one small observational study with a high risk of bias reported data on this outcome. However, supporting evidence regarding vaccine effectiveness against oral HPV infections comes from two sources. Using data from the Costa Rica Trial, Herrero et al. performed a post hoc analysis of the impact of female vaccination with the bivalent vaccine on the prevalence of oral infections 4 years after the first vaccine dose. In this study, vaccine effectiveness against oral HPV 16/18 infections was 93.3% (95% CI 63–100%) [44]. Furthermore, vaccinating adult men with the quadrivalent vaccine was shown to induce neutralizing antibodies in the oral cavity in 65.5% (HPV 18) to 93.2% (HPV 16) of participants [45].

In a number of countries, HPV vaccination of girls and women has already been implemented for nearly a decade. If vaccination coverage in girls and women is high enough, indirect (herd) protection of heterosexual men can be achieved [46]. In the presence of herd protection effects, it is not possible to study the effectiveness of HPV vaccination of men in isolation. Therefore, we examined whether the results of the studies included in our systematic review could have been affected by HPV vaccination programs targeting girls and women in the respective study countries. In fact, five of the included studies were conducted in settings where HPV vaccination of females was implemented [24–26, 31, 34]. However, two of those studies included MSM who do not benefit from such herd protection effects [25, 34]. For the remaining three studies one cannot exclude the possibility that herd protection effects might have slightly influenced the respective study estimates.

## Conclusions

This systematic review shows that the currently available evidence on the efficacy/effectiveness and safety of HPV vaccination in males is limited due to the small number of relevant studies, imprecise estimates and lack of data for some critical outcomes. Vaccine effectiveness drops markedly in individuals who are already infected with the corresponding HPV type. This supports a recommendation for early vaccination of boys with the goal of establishing optimal vaccine-induced protection before the onset of sexual activity. This might not be a realistic option when implementing a program that intends to target only high-risk males such as MSM or HIV-positives. On the other hand, even if the relative vaccine efficacy in such a population with a high prevalence of infection is low, the impact of the vaccine in MSM may be much higher because of the high absolute risk in this group. While the limitations of the evidence base point to a need for further studies in men, it is important to bear in mind that the results of population-based studies might be difficult to interpret in the presence of the above-mentioned herd protection effects. Furthermore, mathematical modelling studies will still be needed to assess the effects of adding males to existing HPV vaccination programs in more detail. The two available studies with vaccination of HPV-naïve males showed high efficacy for reducing dysplasia.

## Additional files

Additional file 1: Search strategy. (DOCX 13 kb)

Additional file 2: List of excluded studies. (DOCX 34 kb)

Additional file 3: GRADE evidence profile. (DOCX 24 kb)
