# Supplementary material for: Efficacy, effectiveness and safety of vaccination against human papillomavirus in males: a systematic review
Source: BMC Med. 2018 Jul 18;16:110. doi: 10.1186/s12916-018-1098-3 (PMC6050686; doi:10.1186/s12916-018-1098-3)
Supplement: Supplementary file 1 — Search strategy. (DOCX 13 kb) [file 12916_2018_1098_MOESM1_ESM.docx]

**Additional File 1:** Search strategy

Search in Medline, Embase and Cochrane Central Register of Controlled Trials (full-text search; date of last search: 18 April 2017):

#1 papillomaviridae

#2 HPV

#3 papillomavirus

#4 male

#5 man

#6 men

#7 boy*

#8 vaccin*

#9 immunis*

#10 gardasil

#11 silgard

#12 #1 OR #2 OR #3

#13 #4 OR #5 OR #6 OR #7

#14 #8 OR #9 OR #10 OR #11

#15 #12 AND #13 AND #14

(steps #10 and #11 were substituted by “cervarix” in a subsequent search)
