# Supplementary material for: Efficacy, effectiveness and safety of vaccination against human papillomavirus in males: a systematic review
Source: BMC Med. 2018 Jul 18;16:110. doi: 10.1186/s12916-018-1098-3 (PMC6050686; doi:10.1186/s12916-018-1098-3)
Supplement: Supplementary file 2 — List of excluded studies. (DOCX 34 kb) [file 12916_2018_1098_MOESM2_ESM.docx]

**Additional File 2:** List of excluded studies

1. NCT01862874: Efficacy and tolerability study of V501 in Japanese males (V501-122). Secondary NCT01862874: Efficacy and tolerability study of V501 in Japanese males (V501-122). [www.clinicaltrials.gov](http://www.clinicaltrials.gov).

2. NCT02003508: Prevalence of genital HPV infection in males following introduction of universal male HPV vaccination (Impress). Secondary NCT02003508: Prevalence of genital HPV infection in males following introduction of universal male HPV vaccination (Impress). [www.clinicaltrials.gov](http://www.clinicaltrials.gov).

3. Reisinger KS, Block SL, Lazcano-Ponce E, et al. Safety and persistent immunogenicity of a quadrivalent human papillomavirus types 6, 11, 16, 18 L1 virus-like particle vaccine in preadolescents and adolescents: a randomized controlled trial. The Pediatric infectious disease journal 2007;**26**(3):201-9.

4. Centers for Disease C, Prevention. Syncope after vaccination--United States, January 2005-July 2007. MMWR Morbidity and mortality weekly report 2008;**57**(17):457-60.

5. Hillman RJ, Hillman RJ. The effficacy of quadrivalent HPV (types 6/11/16/18) vaccine against HPV-related genital disease and infection in HIV negative young men. Sexual Health 2009;**6**(4):357.

6. Block SL, Brown DR, Chatterjee A, et al. Clinical trial and post-licensure safety profile of a prophylactic human papillomavirus (types 6, 11, 16, and 18) l1 virus-like particle vaccine. The Pediatric infectious disease journal 2010;**29**(2):95-101.

7. Elbasha EH, Dasbach EJ. Impact of vaccinating boys and men against HPV in the United States, 2010:6858-67.

8. Levin MJ, Moscicki AB, Song LY, et al. Safety and immunogenicity of a quadrivalent human papillomavirus (types 6, 11, 16, and 18) vaccine in HIV-infected children 7 to 12 years old. Journal of acquired immune deficiency syndromes (1999) 2010;**55**(2):197-204.

9. Yancey AM, Pitlick JM, Forinash AB, et al. The prophylactic role for the human papillomavirus quadrivalent vaccine in males. The Annals of pharmacotherapy 2010;**44**(7-8):1314-8.

10. Erratum: Efficacy of quadrivalent HPV vaccine against HPV infection and disease in males (New England Journal of Medicine (2011) 364 (401-411)). New England Journal of Medicine 2011;**364**(15):1481.

11. Dietz CA, Nyberg CR, Dietz CA, et al. Genital, oral, and anal human papillomavirus infection in men who have sex with men. The Journal of the American Osteopathic Association 2011;**111**(3 Suppl 2):19-25.

12. Garnock-Jones KP, Giuliano AR. Quadrivalent Human Papillomavirus (HPV) types 6, 11, 16, 18 vaccine: For the prevention of genital warts in males. Drugs 2011;**71**(5):591-602.

13. Goldstone S, Goldstone S. Efficacy of the quadrivalent hpv vaccine to prevent anal intraepithelial neoplasia among young men who have sex with men. Sexually Transmitted Infections 2011;**87**:352.

14. Haupt RM, Sings HL, Haupt RM, et al. The efficacy and safety of the quadrivalent human papillomavirus 6/11/16/18 vaccine gardasil. The Journal of adolescent health : official publication of the Society for Adolescent Medicine 2011;**49**(5):467-75.

15. Khalid JM, Amelio JM, Carroll SM, et al. The broader burden of HPV-related disease in England: A preliminary analysis. Value in Health 2011;**14**(7):437.

16. Lepage AK, McIntyre RC, Kennedy SE, et al. Safety and reactogenicity of the human papilloma virus vaccine in kidney transplant patients. Immunology and Cell Biology 2011;**89**(7):22.

17. Lescaille G, Descroix V, Azérad J, et al. Papillomavirus et cancers des VADS

[Papillomavirus and head and neck cancer]. Revue de stomatologie et de chirurgie maxillo-faciale 2011;**112**(3):160-3.

18. Moreira ED, Palefsky JM, Giuliano AR, et al. Safety and reactogenicity of a quadrivalent human papillomavirus (types 6, 11, 16, 18) L1 viral-like-particle vaccine in older adolescents and young adults. Human vaccines 2011;**7**(7):768-75.

19. Pomfret TC, Gagnon Jm Jr, Gilchrist AT, et al. Quadrivalent human papillomavirus (HPV) vaccine: a review of safety, efficacy, and pharmacoeconomics. Journal of clinical pharmacy and therapeutics 2011;**36**(1):1-9.

20. Bauer HM, Wright G, Chow J, et al. Evidence of human papillomavirus vaccine effectiveness in reducing genital warts: an analysis of California public family planning administrative claims data, 2007-2010. American journal of public health 2012;**102**(5):833-5.

21. Blomberg M, Friis S, Munk C, et al. Genital warts and risk of cancer: a Danish study of nearly 50 000 patients with genital warts. The Journal of infectious diseases 2012;**205**(10):1544-53.

22. Chaux A, Cubilla AL, Chaux A, et al. The role of human papillomavirus infection in the pathogenesis of penile squamous cell carcinomas. Seminars in diagnostic pathology 2012;**29**(2):67-71.

23. Goldstone SE, Vuocolo S, Goldstone SE, et al. A prophylactic quadrivalent vaccine for the prevention of infection and disease related to HPV-6, -11, -16 and -18. Expert review of vaccines 2012;**11**(4):395-406.

24. Hillman RJ, Giuliano AR, Palefsky JM, et al. Immunogenicity of the quadrivalent human papillomavirus (type 6/11/16/18) vaccine in males 16 to 26 years old. Clinical and vaccine immunology : CVI 2012;**19**(2):261-7.

25. Li R, Li Y, Radley D, et al. Safety and immunogenicity of a vaccine targeting human papillomavirus types 6, 11, 16 and 18: a randomized, double-blind, placebo-controlled trial in Chinese males and females. Vaccine 2012;**30**(28):4284-91.

26. Read TR, Hocking JS, Vodstrcil LA, et al. Oral human papillomavirus in men having sex with men: risk-factors and sampling. PloS one 2012;**7**(11):49324.

27. Sanders AE, Slade GD, Patton LL, et al. National prevalence of oral HPV infection and related risk factors in the U.S. adult population. Oral diseases 2012;**18**(5):430-41.

28. Sethi S, Ali-Fehmi R, Franceschi S, et al. Characteristics and survival of head and neck cancer by HPV status: a cancer registry-based study. International journal of cancer 2012;**131**(5):1179-86.

29. Willett CG, Willett CG. Palefsky J, Giuliano A, Gladstone S, et al. HPV vaccine against anal HPV infection and anal intraepithelial neoplasia. New Eng J Med 2011; 365:1576-85. International Journal of Radiation Oncology Biology Physics 2012;**82**(4):1301.

30. Bosnjak Z, Peric M, Krizan IR, et al. Prevalence and genotype distribution of high-risk human papillomavirus (HR HPV) in male genital samples of Osijek-Baranja County. Collegium antropologicum 2013;**37**(4):1203-8.

31. Goldstone SE, Jessen H, Palefsky JM, et al. Quadrivalent HPV vaccine efficacy against disease related to vaccine and non-vaccine HPV types in males. Vaccine 2013;**31**(37):3849-55.

32. Kumar D, Unger ER, Panicker G, et al. Immunogenicity of quadrivalent human papillomavirus vaccine in organ transplant recipients. American journal of transplantation : official journal of the American Society of Transplantation and the American Society of Transplant Surgeons 2013;**13**(9):2411-7.

33. Larsen HK, Kofoed K, Sand C, et al. Den HPV-relaterede sygdomsbyrde hos mænd er stor og kan forebygges. Ugeskrift for Laeger 2013;**175**(6):349-53.

34. Larsen HK, Larsen HK. HPV-infektion og anal dysplasi hos mænd, der har sex med mænd, er hyppig og bør forebygges. Ugeskrift for Laeger 2013;**175**(6):345-49.

35. Lawton MD, Nathan M, Asboe D, et al. HPV vaccination to prevent anal cancer in men who have sex with men. England, 2013:342-3.

36. Macartney KK, Chiu C, Georgousakis M, et al. Safety of human papillomavirus vaccines: a review. Drug safety 2013;**36**(6):393-412.

37. Nordfors C, Grün N, Haeggblom L, et al. Oral human papillomavirus prevalence in high school students of one municipality in Sweden. Scandinavian journal of infectious diseases 2013;**45**(11):878-81.

38. Nsouli-Maktabi H, Ludwig SL, Yerubandi UD, et al. Incidence of genital warts among U.S. service members before and after the introduction of the quadrivalent human papillomavirus vaccine. MSMR 2013;**20**(2):17-20.

39. Staltari O, Cilurzo F, Caroleo B, et al. Annual report on adverse events related with vaccines use in Calabria (Italy): 2012. Journal of Pharmacology and Pharmacotherapeutics 2013;**4**(suppl.1):61-65.

40. Swedish K, Goldstone SE. Prevention of anal condyloma with quadrivalent human papillomavirus vaccination of older men who have sex with men: A nonconcurrent cohort study. Journal of general internal medicine 2013;**28**:155.

41. Blitshteyn S, Blitshteyn S. Postural tachycardia syndrome following human papillomavirus vaccination. European Journal of Neurology 2014;**21**(1):135-39.

42. Cockerill CC, Orvidas LJ, Moore EJ, et al. Human papillomavirus infection in benign tonsil swabs and tonsillectomy specimens. Otolaryngology - Head and Neck Surgery (United States) 2014;**151**(1):41-42.

43. Cranston R, Yang M, Paczuski P, et al. Baseline Data of a Phase 3 Trial of the Quadrivalent HPV Vaccine in HIV+ Males and Females: ACTG 5298. Topics in antiviral medicine 2014;**22**(e-1):364.

44. Ferris D, Samakoses R, Block SL, et al. Long-term study of a quadrivalent human papillomavirus vaccine. Pediatrics 2014;**134**(3):657-65.

45. Giacomet V, Penagini F, Trabattoni D, et al. Safety and immunogenicity of a quadrivalent human papillomavirus vaccine in HIV-infected and HIV-negative adolescents and young adults. Vaccine 2014;**32**(43):5657-61.

46. Gomez-Lobo V, Whyte T, Kaufman S, et al. Immunogenicity of a prophylactic quadrivalent human papillomavirus L1 virus-like particle vaccine in male and female adolescent transplant recipients. Pediatric transplantation 2014;**18**(3):310-5.

47. Langer-Gould A, Qian L, Tartof SY, et al. Vaccines and the risk of multiple sclerosis and other central nervous system demyelinating diseases. JAMA neurology 2014;**71**(12):1506-13.

48. Markowitz LE, Dunne EF, Saraiya M, et al. Human papillomavirus vaccination: recommendations of the Advisory Committee on Immunization Practices (ACIP). MMWR Recommendations and reports : Morbidity and mortality weekly report Recommendations and reports / Centers for Disease Control 2014;**63**(RR-05):1-30.

49. Ojha RP, Jackson BE, Tota JE, et al. Guillain-Barre syndrome following quadrivalent human papillomavirus vaccination among vaccine-eligible individuals in the United States. Human vaccines & immunotherapeutics 2014;**10**(1):232-7.

50. Ozkan TA, Coskuner E, Karakose A, et al. Impact of the quadrivalent HPV vaccine for men who exposed to HPV infection. Journal of urology 2014;**191**(4 SUPPL. 1):161.

51. Pellegrino P, Perrone V, Pozzi M, et al. Immunologic Research 2014;**61**(1-2):90-96.

52. Schurink-van 't Klooster TM, De Ridder MAJ, Kemmeren JM, et al. Determination of a possible association between human papillomavirus (HPV) vaccination and migraine. Pharmacoepidemiology and Drug Safety 2014;**23**:151.

53. Schurink-Van't Klooster TM, de Ridder MAJ, Kemmeren JM, et al. Examining a possible association between human papilloma virus (HPV) vaccination and migraine: Results of a cohort study in the Netherlands. European Journal of Pediatrics 2014;**174**(5):641-49.

54. Söderlund-Strand A, Uhnoo I, Dillner J, et al. Change in population prevalences of human papillomavirus after initiation of vaccination: The high-throughput HPV monitoring study. Cancer Epidemiology Biomarkers and Prevention 2014;**23**(12):2757-64.

55. Wierzbicka M, Józefiak A, Jackowska J, et al. HPV vaccination in head and neck HPV-related pathologies. Otolaryngologia polska = The Polish otolaryngology 2014;**68**(4):157-73.

56. Ali H, O'Connor CC, Callander D, et al. The impact of HPV vaccination on genital warts in aboriginal australians: Analysis of national data. Sexually Transmitted Infections 2015;**91**:78.

57. Castellsague X, Giuliano AR, Goldstone S, et al. Immunogenicity and safety of the 9-valent HPV vaccine in men. Vaccine 2015;**33**(48):6892-901.

58. Drolet M, Bénard É, Boily MC, et al. Population-level impact and herd effects following human papillomavirus vaccination programmes: a systematic review and meta-analysis. The Lancet Infectious diseases 2015;**15**(5):565-80.

59. Giuliano AR, Isaacs-Soriano K, Torres BN, et al. Immunogenicity and safety of Gardasil among mid-adult aged men (27-45 years)--The MAM Study. Vaccine 2015;**33**(42):5640-6.

60. Kosalaraksa P, Mehlsen J, Vesikari T, et al. An Open-Label, Randomized Study of a 9-Valent Human Papillomavirus Vaccine Given Concomitantly with Diphtheria, Tetanus, Pertussis and Poliomyelitis Vaccines to Healthy Adolescents 11-15 Years of Age. Pediatric infectious disease journal 2015;**34**(6):627-34.

61. Krone B, Grange JM, Krone B, et al. Vaccination, infection, and the risk for multiple sclerosis. United States, 2015:480.

62. Mahajan D, Dey A, Cook J, et al. Surveillance of adverse events following immunisation in Australia annual report, 2013. Communicable diseases intelligence quarterly report 2015;**39**(3):369-86.

63. Mariani L, Vici P, Suligoi B, et al. Early Direct and Indirect Impact of Quadrivalent HPV (4HPV) Vaccine on Genital Warts: a Systematic Review. Advances in Therapy 2015;**32**(1):10-30.

64. Perkins RB, Legler A, Hanchate A, et al. Trends in Male and Female Genital Warts Among Adolescents in a Safety-Net Health Care System 2004-2013: Correlation With Introduction of Female and Male Human Papillomavirus Vaccination. Sexually transmitted diseases 2015;**42**(12):665-8.

65. Ristori G, Mechelli R, Salvetti M, et al. Association between vaccines and neuroinflammation: time, risks, and benefits. JAMA neurology 2015;**72**(5):605.

66. Schurink-Van't Klooster TM, de Ridder MA, Kemmeren JM, et al. Examining a possible association between human papilloma virus (HPV) vaccination and migraine: results of a cohort study in the Netherlands. European journal of pediatrics 2015;**174**(5):641-9.

67. Tan P, Wang X, Wei S, et al. Efficacy and safety of prophylactic human papillomavirus vaccination in healthy males: A meta-analysis. Reviews in Medical Microbiology 2015;**26**(4):143-53.

68. Van Damme P, Olsson SE, Block S, et al. Immunogenicity and Safety of a 9-Valent HPV Vaccine. Pediatrics 2015;**136**(1):28-39.

69. Vichnin M, Bonanni P, Klein NP, et al. An Overview of Quadrivalent Human Papillomavirus Vaccine Safety: 2006 to 2015. The Pediatric infectious disease journal 2015;**34**(9):983-91.

70. Chandler EL, Ding L, Widdice L, et al. Epidemiology of anogenital human papillomavirus (HPV) among 13-26 year-old young men after HPV vaccine introduction. Journal of Adolescent Health 2016;**58**(2):117-18.

71. Clark LR, Luxembourg AT, Clark LR, et al. Clinical trial experience with the 9-valent HPV vaccine by race/ethnicity: A combined analysis from seven phase iii clinical studies. Journal of Adolescent Health 2016;**58**(2):118.

72. Crawford NW, Hodgson K, Gold M, et al. Adverse Events Following HPV Immunisation in Australia: Establishment of a Clinical Network. Human vaccines & immunotherapeutics 2016:0.

73. Das R, Das R. Effectiveness, immunogenicity, and safety of gardasiltm in pre-adolescents and adolescents-10 years of follow-up. Journal of Adolescent Health 2016;**58**(2):10.

74. Kahn JA, Rudy BJ, Xu J, et al. Prevalence and risk factors for oral DNA tumor viruses in HIV-infected youth. Journal of medical virology 2016;**88**(11):1944-52.

75. King EM, Oomeer S, Gilson R, et al. Oral Human Papillomavirus Infection in Men Who Have Sex with Men: A Systematic Review and Meta-Analysis. PloS one 2016;**11**(7):0157976.

76. Moreira Ed Jr, Block SL, Ferris D, et al. Safety Profile of the 9-Valent HPV Vaccine: A Combined Analysis of 7 Phase III Clinical Trials. Pediatrics 2016;**138**(2).

77. Naleway AL, Crane B, Smith N, et al. Absence of venous thromboembolism risk following quadrivalent human papillomavirus vaccination, Vaccine Safety Datalink, 2008-2011. Vaccine 2016;**34**(1):167-71.

78. Pinto LA, Kemp TJ, Torres BN, et al. THE QUADRIVALENT HPV VACCINE INDUCES HPV-SPECIFIC ANTIBODIES AT THE ORAL CAVITY: RESULTS FROM THE MID-ADULT MALE VACCINE TRIAL-THE MAM TRIAL. The Journal of infectious diseases 2016.

79. Van Damme P, Meijer CJ, Kieninger D, et al. A phase III clinical study to compare the immunogenicity and safety of the 9-valent and quadrivalent HPV vaccines in men. Vaccine 2016;**34**(35):4205-12.
