# Supplementary material for: Efficacy, effectiveness and safety of vaccination against human papillomavirus in males: a systematic review
Source: BMC Med. 2018 Jul 18;16:110. doi: 10.1186/s12916-018-1098-3 (PMC6050686; doi:10.1186/s12916-018-1098-3)
Supplement: Supplementary file 3 — GRADE evidence profile. (DOCX 24 kb) [file 12916_2018_1098_MOESM3_ESM.docx]

**Additional File 3:** GRADE evidence profile

| **Quality assessment** | | | | | | | **No of patients** | | **Effect** | | **Quality** | **Importance** |
| --- | --- | --- | --- | --- | --- | --- | --- | --- | --- | --- | --- | --- |
|  |  |  |  |  |  |  |  |  |  |  |  |  |
| **No of studies** | **Design** | **Risk of bias** | **Inconsistency** | **Indirectness** | **Imprecision** | **Publication bias** | **Vaccination against HPV** | **No vaccination** | **Relative (95% CI)** | **Absolute** |  |  |
| **incident oral infection with high-risk HPV type (assessed with: HPV-16 and/or HPV-18)** | | | | | | | | | | | | |
| 1 | observational studies | serious^1^ | no serious inconsistency | no serious indirectness | serious^2^ | none | 0/23  (0%) | 7/142  (4.9%) | RR 0.40 (0.02 to 6.73) | 30 fewer per 1000 (from 48 fewer to 282 more) | ⊕OOO VERY LOW | CRITICAL |
|  |  |  |  |  |  |  |  | 10% |  | 60 fewer per 1000 (from 98 fewer to 573 more) |  |  |
|  |  |  |  |  |  |  |  | 20% |  | 120 fewer per 1000 (from 196 fewer to 1000 more) |  |  |
| **incident anogenital infection with high-risk HPV type (assessed with: HPV-16)** | | | | | | | | | | | | |
| 1 | randomised trials | no serious risk of bias | no serious inconsistency | no serious indirectness | serious^2^ | none | 189/4070  (4.6%)^3^ | 259/4014  (6.5%)^3^ | RR 0.72 (0.59 to 0.87) | 18 fewer per 1000 (from 8 fewer to 26 fewer) | ⊕⊕⊕O MODERATE | CRITICAL |
|  |  |  |  |  |  |  |  | 1% |  | 3 fewer per 1000 (from 1 fewer to 4 fewer) |  |  |
|  |  |  |  |  |  |  |  | 10% |  | 28 fewer per 1000 (from 13 fewer to 41 fewer) |  |  |
| **persisting oral infection with high-risk HPV type (follow-up mean 2.1 years)** | | | | | | | | | | | | |
| 1 | randomised trials | no serious risk of bias | no serious inconsistency | no serious indirectness | serious^2^ | none | 1/236  (0.42%) | 8/236  (3.4%) | RR 0.12 (0.02 to 0.98) | 30 fewer per 1000 (from 1 fewer to 33 fewer) | ⊕⊕⊕O MODERATE | CRITICAL |
|  |  |  |  |  |  |  |  | 1% |  | 9 fewer per 1000 (from 0 fewer to 10 fewer) |  |  |
|  |  |  |  |  |  |  |  | 6% |  | 53 fewer per 1000 (from 1 fewer to 59 fewer) |  |  |
| **persisting anogenital infection with high-risk HPV type (assessed with: HPV-16)** | | | | | | | | | | | | |
| 1 | randomised trials | no serious risk of bias | no serious inconsistency | no serious indirectness | serious^2^ | none | 71/4199  (1.7%)^3^ | 131/4112  (3.2%)^3^ | RR 0.53 (0.39 to 0.71) | 15 fewer per 1000 (from 9 fewer to 19 fewer) | ⊕⊕⊕O MODERATE | CRITICAL |
|  |  |  |  |  |  |  |  | 1% |  | 5 fewer per 1000 (from 3 fewer to 6 fewer) |  |  |
|  |  |  |  |  |  |  |  | 6% |  | 28 fewer per 1000 (from 17 fewer to 37 fewer) |  |  |
| **condylomata acuminata** | | | | | | | | | | | | |
| 1 | randomised trials | no serious risk of bias | no serious inconsistency | no serious indirectness | no serious imprecision | none | 24/4635  (0.52%)^3^ | 72/4559  (1.6%)^3^ | RR 0.33 (0.2 to 0.53) | 11 fewer per 1000 (from 7 fewer to 13 fewer) | ⊕⊕⊕⊕ HIGH | IMPORTANT |
|  |  |  |  |  |  |  |  | 5% |  | 34 fewer per 1000 (from 24 fewer to 40 fewer) |  |  |
|  |  |  |  |  |  |  |  | 10% |  | 67 fewer per 1000 (from 47 fewer to 80 fewer) |  |  |
| **anal intraepithelial neoplasia (AIN) grade 2** | | | | | | | | | | | | |
| 1 | randomised trials | no serious risk of bias | no serious inconsistency | no serious indirectness | serious^2^ | none | 11/668  (1.6%)^3^ | 29/671  (4.3%)^3^ | RR 0.38 (0.17 to 0.79) | 27 fewer per 1000 (from 9 fewer to 36 fewer) | ⊕⊕⊕O MODERATE | CRITICAL |
|  |  |  |  |  |  |  |  | 1% |  | 6 fewer per 1000 (from 2 fewer to 8 fewer) |  |  |
|  |  |  |  |  |  |  |  | 8% |  | 50 fewer per 1000 (from 17 fewer to 66 fewer) |  |  |
| **anal intraepithelial neoplasia (AIN) grade 3 or carcinoma** | | | | | | | | | | | | |
| 1 | randomised trials | no serious risk of bias | no serious inconsistency | no serious indirectness | very serious^4^ | none | 10/666  (1.5%)^3^ | 19/673  (2.8%)^3^ | RR 0.53 (0.22 to 1.2) | 13 fewer per 1000 (from 22 fewer to 6 more) | ⊕⊕OO LOW | CRITICAL |
|  |  |  |  |  |  |  |  | 1% |  | 5 fewer per 1000 (from 8 fewer to 2 more) |  |  |
|  |  |  |  |  |  |  |  | 6% |  | 28 fewer per 1000 (from 47 fewer to 12 more) |  |  |
| **penile intraepithelial neoplasia grade 2 or 3** | | | | | | | | | | | | |
| 1 | randomised trials | no serious risk of bias | no serious inconsistency | serious^5^ | very serious^4^ | none | 3/4663  (0.06%)^3^ | 2/4628  (0.04%)^3^ | RR 1.49 (0.17 to 17.8) | 0 more per 1000 (from 0 fewer to 7 more) | ⊕OOO VERY LOW | IMPORTANT |
|  |  |  |  |  |  |  |  | 0.5% |  | 2 more per 1000 (from 4 fewer to 84 more) |  |  |
|  |  |  |  |  |  |  |  | 1% |  | 5 more per 1000 (from 8 fewer to 168 more) |  |  |
| **squamous cell carcinoma of the head and neck region** | | | | | | | | | | | | |
| 0 | No evidence available |  |  |  |  | none | - | - | - | - |  | IMPORTANT |
|  |  |  |  |  |  |  |  | 0% |  | - |  |  |
| **epithelial neoplasia related to squamous cell carcinoma of the head and neck region** | | | | | | | | | | | | |
| 0 | No evidence available |  |  |  |  | none | - | - | - | - |  | IMPORTANT |
|  |  |  |  |  |  |  |  | 0% |  | - |  |  |
| **severe adverse events** | | | | | | | | | | | | |
| 1 | randomised trials | no serious risk of bias | no serious inconsistency | no serious indirectness | serious^2^ | none | 8/2020  (0.4%) | 11/2029  (0.54%) | RR 0.73 (0.25 to 1.99) | 1 fewer per 1000 (from 4 fewer to 5 more) | ⊕⊕⊕O MODERATE | CRITICAL |
|  |  |  |  |  |  |  |  | 1% |  | 3 fewer per 1000 (from 7 fewer to 10 more) |  |  |
|  |  |  |  |  |  |  |  | 5% |  | 13 fewer per 1000 (from 38 fewer to 49 more) |  |  |

^1^ estimate not adjusted for confounders
^2^ wide 95%CI
^3^ person-years as denominator
^4^ very wide 95%CI including large benefits and risks
^5^ outcome comprises penile, perineal and perianal neoplasia
